# Development and Validation of a RP-HPLC Method for Estimation of Montelukast Sodium in Bulk and in Tablet Dosage Form

**DOI:** 10.4103/0250-474X.65023

**Published:** 2010

**Authors:** R. M. Singh, P. K. Saini, S. C. Mathur, G. N. Singh, B. Lal

**Affiliations:** Research and Development Division, Indian Pharmacopoeia Commission, Sector-23, Rajnagar, Ghaziabad-201 002, India; 1 Department of Pharmaceutical Analysis, Faculty of Pharmacy, Vinayaka Mission University, Salem-636 015, India

**Keywords:** Montelukast, HPLC, method development and validation

## Abstract

The present work describes a simple, precise and accurate HPLC method for estimation of montelukast sodium in bulk and in tablet dosage form. The separation was achieved by using octadecylsilane column (C18) and acetonitrile:1 mM sodium acetate adjusted to pH 6.3 with acetic acid in proportion of 90:10 v/v as mobile phase, at a flow rate of 1.5 ml/min. Detection was carried out at 285 nm. The retention time of montelukast sodium was found to be 3.4 min. The limit of detection was found 1.31 µg/ml and limit of quantification 3.97 µg/ml. The accuracy and reliability of the proposed method was ascertained by evaluating various validation parameters like linearity (1-100 µg/ml), precision, accuracy and specificity according to ICH guidelines. The proposed method provides an accurate and precise quality control tool for routine analysis of montelukast sodium in bulk and in tablet dosage form.

Montelukast sodium is chemically (R-(E))-1-(((1-(3-(2-(7-chloro-2-quinolinyl) ethenyl)phenyl)-3(2-(1-hydroxy-1-methylethyl)phenyl)propyl)thio)methyl)cyclopropaneacetic acid, monosodium salt[1–2]. Montelukast sodium primarily used for the treatment of asthma in children and adults[3–5]. It is a potent selective inhibitor of leukotriene D4 (LTD4) at the cysteinyl leukotriene receptor cysLT1[6–7]. Literature survey reveals that liquid chromatography with fluorescence detector[8], stereoselective high performance liquid chromatography (HPLC) for montelukast and its S-enantiomer[9], column switching HPLC with fluorescence detector[10], semi-automated 96-well protein precipitation[11], HPLC with derivative spectroscopy[12], pressurized liquid extraction followed by HPLC[13] and LC-MS methods[14–16] have been reported for the estimation of montelukast sodium. The present study illustrates development and validation of a simple, accurate and precise procedure for determination of montelukast sodium by RP-HPLC in bulk and in tablet dosage form.

Montelukast sodium working standard was obtained from Vitalife Laboratories (A division of Arch Pharma lab) Gurgaon, Haryana, India. Montair Tablets (montelukast sodium tablets, 10 mg) was purchased from a local pharmacy store. All chemicals and reagents used were of HPLC grade and purchased from E. Merck Chemicals Corporation Ltd. Mumbai, India.

A waters HPLC system containing 600 controller, inline degasser, 717 plus auto-sampler, 486 tunable absorbance detector and temperature control device with operating software Millennium 32 were used during the study. A sunfire C_18_ column (250×4.6 mm, 5 µm particle size) was used as stationary phase. The mobile phase was optimized with acetonitrile and 1 mM sodium acetate buffer (adjusted to pH 6.3 with acetic acid), in the proportion of 90:10 v/v, UV detection was carried out at 285 nm with a flow rate of 1.5 ml/min.

About 10 mg of montelukast sodium standard was weighed accurately and transferred to 100 ml volumetric flask. The volume was made up to mark with the mobile phase to obtain a concentration of 100 µg/ml. Further dilutions were made to obtain the concentration in the range of 1-100 µg/ml of montelukast sodium. For analysis in tablet dosage form, twenty tablets were weighed. The tablets were finely powdered and powder equivalent to 10 mg of montelukast sodium was accurately weighed and transferred into a 100 ml volumetric flask. The volume was made up to mark with the mobile phase to obtain a concentration of 100 µg/ml. Further dilutions were made to obtain a concentration of 10 µg/ml and filtered through 0.45 µm membrane filter.

The system suitability was checked by injecting 20 µl of standard solution and found the results within the range. The relative standard deviation on five replicate injections was obtained 0.5%, tailing factor 1.25, and the column efficiency 1278 theoretical plates.

Twenty microlitres of standard and sample solutions were separately injected on HPLC system. From the peak area of montelukast the amount of drugs in the sample were computed. The analysis was repeated in triplicate. The % assay of the bulk was found to be 100.3±0.60 and for tablet dosage form 99.51±1.76 of the labelled claim.

The developed method was validated in terms of specificity, linearity, accuracy, limit of detection, limit of quantification, intra-day and inter-day precision and robustness for the assay of montelukast sodium as per ICH guidelines[17].

Specificity was studied for the examination of the presence of interfering components. Montelukast standard solution of 10 µg/ml was injected and none of the impurities were interfering in its assay. The retention time obtained by HPLC for montelukast is about 3.4 min as shown in fig. 1.

**Fig. 1:**
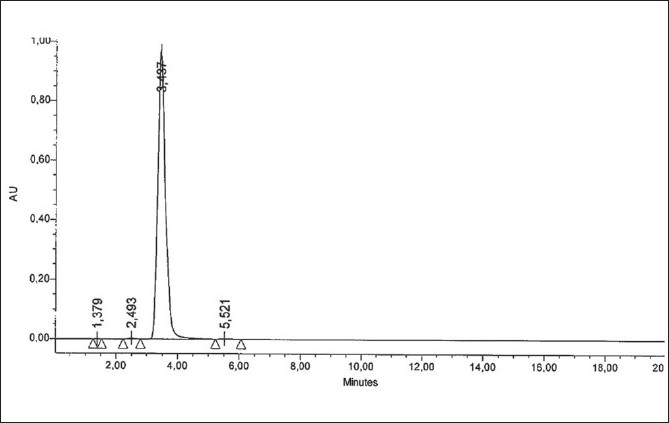
A typical chromatogram of montelukast sodium standard (10 µg/ml)

Linearity was studied by preparing standard solutions of montelukast at different concentration levels. The linearity ranges were found in the range of 1-100 µg/ml. The standard calibration curve was generated using regression analysis with Microsoft excel. The assay was judged to be linear as the correlation coefficient was greater than 0.995 by the least-square method as shown in Table 1.

**TABLE 1:** VALIDATION PARAMETERS OF THE PROPOSED METHOD

Parameter	Montelukast sodium
Linearity range (µg/ml)	1-100
Correlation coefficient (r)	0.9990
Regression equation (y=mx+c) Slope (m)	7382
Intercept (c)	34277
Limit of detection (LOD) (µg/ml)	1.31
Limit of quantification (LOQ) (µg/ml)	3.97

Recovery studies of the drug were carried out for the accuracy parameter at three different concentration levels i.e. multiple level recovery studies. A known amount of montelukast standard was added into pre-analysed sample and subjected to the proposed HPLC method. Percentage recovery was found to be within the limits as listed in Table 2.

**TABLE 2:** RECOVERY STUDIES OF MONTELUKAST IN TABLET

Label claim mg/ tablet	Total amount added (mg)	Amount Recovered* (mg)±SD	% Recovery ±SD
Montelukast	8.0	7.94±0.06	99.25±0.06
sodium	10.00	9.91±0.01	99.10±0.02
10	12.00	11.85±0.12	98.75±0.13

* Each value is a mean±standard deviation of three determinations

Precision was studied to find out intra and inter day variations in the test methods of montelukast in the concentrationrange of 5-50 µg/ml for three times on the same day and different day. Precision was determined by analysing corresponding standard daily for a period of three days. The inter-day and intra-day precision obtained was % RSD (<2) indicates that the proposed method is quite precise and reproducible as shown in Table 3.

**TABLE 3:** INTRA-DAY AND INTER-DAY PRECISION OF MONTELUKAST (N = 3)

Nominal concentration (µg/ml)	Day 1			Day 2			Day 3		
	Mean	SD	%RSD	Mean	SD	%RSD	Mean	SD	%RSD
5	4.95	0.15	0.81	4.89	0.14	0.83	4.86	0.11	1.14
10	10.03	0.64	0.57	9.98	0.58	0.97	9.91	0.84	1.10
50	49.69	0.77	0.54	49.62	0.67	0.55	49.45	0.67	0.55

The Limit of Detection (LOD) and Limit of Quantitation (LOQ) was calculated based on the standard deviation (SD) of the response and the slope (S) of the calibration curve at levels approximating the LOD and LOQ, LOD= 3.3 (SD/S) and LOQ= 10 (SD/S) is shown in Table 1. Robustness was done by small changes in the chromatographic conditions like mobile phase, flow rate etc. and found to be unaffected.

A simple, accurate, fast and precise isocratic reverse phase high performance liquid chromatographic method has been developed for the determination of montelukast sodium in bulk and in tablet dosage form. The developed method was found to be simple and have short run time which makes the method rapid. The results of the study indicate that the proposed HPLC method is simple, precise, accurate and less time consuming.
